# Workers’ Psychological Distress During the Early Months of the COVID-19 Pandemic in Brazil: A Cross-Sectional Study

**DOI:** 10.3390/bs15030358

**Published:** 2025-03-13

**Authors:** Melissa Spröesser Alonso, Maria Cristina Pereira Lima, Adriano Dias, Hélio Rubens de Carvalho Nunes, Carlos Ruiz-Frutos, Javier Fagundo-Rivera, Juan Gómez-Salgado, João Marcos Bernardes

**Affiliations:** 1 Graduate Program in Collective/Public Health, Botucatu Medical School, São Paulo State University (UNESP), Botucatu 18618687, São Paulo, Brazil; 2 Department of Neurosciences and Mental Health, Botucatu Medical School, São Paulo State University (UNESP), Botucatu 18618687, São Paulo, Brazil; 3 Department of Public Health, Botucatu Medical School, São Paulo State University (UNESP), Botucatu 18618687, São Paulo, Brazil; 4 Graduate Program in Nursing Academic Master’s and Doctoral Programs, Botucatu Medical School, São Paulo State University (UNESP), Botucatu 18618687, São Paulo, Brazil; 5 Department of Sociology, Social Work and Public Health, Faculty of Labour Sciences, University of Huelva, 21007 Huelva, Spain; 6 Safety and Health Postgraduate Programme, Universidad Espíritu Santo, Guayaquil 092301, Ecuador; 7 Centro Universitario de Enfermería Cruz Roja, University of Seville, 41009 Sevilla, Spain

**Keywords:** COVID-19, preventive medicine, psychological distress, epidemiology, mental health, socioeconomic aspects of health

## Abstract

The COVID-19 pandemic significantly impacted the mental health of workers. This study aimed to assess the prevalence of psychological distress among Brazilian workers during the early months of the pandemic and explore its associated factors. A cross-sectional study was conducted using an online questionnaire administered to 2903 Brazilian workers, including 1752 non-healthcare workers (NHCWs) and 1151 healthcare workers (HCWs), between April and May 2020. Snowball sampling was employed for participant recruitment, and the research questionnaire was adapted for use with the Brazilian population through a process of translation and cultural adaptation, based on an instrument initially created and validated for use in Spain. Differences between NHCWs and HCWs were tested using the chi-square or Fisher’s Exact test and Mann–Whitney test followed by effect size measurement. Multiple linear regression models were used to analyze the association between psychological distress and the predictor variables. Psychological distress was observed in 72.6% (95% CI 70.1–74.2%) of the participants, with no significant difference observed between NHCWs and HCWs. Although 32 variables showed statistically significant differences between NHCWs and HCWs, only 7 demonstrated clinical–epidemiological relevance, primarily related to occupational factors. Work-related stress was positively associated with psychological distress, but this relationship diminished in the absence of family members infected with COVID-19. Conversely, a higher sense of coherence was protective against psychological distress, although this effect weakened in the absence of family members infected with COVID-19. The study highlights the high prevalence of psychological distress among Brazilian workers during the pandemic’s early months. Work stress played a significant role, while sense of coherence appeared to mitigate mental health challenges. These findings highlight the need for targeted mental health interventions, particularly for workers facing both professional and family-related stressors during crises.

## 1. Introduction

The COVID-19 pandemic, caused by the SARS-CoV-2 virus, originated in Wuhan, China, in December 2019. The virus rapidly spread beyond China’s borders, leading to widespread transmission across multiple countries. On 11 March 2020, the World Health Organization officially declared COVID-19 a global pandemic (Huang et al., 2020; Carvalho et al., 2021). In Brazil, the first confirmed case of COVID-19 was reported in late February 2020, in São Paulo. The virus quickly disseminated, leading to significant community transmission and a steep increase in cases. By 30 May 2020, Brazil had become one of the global epicenters of the pandemic, with over 500,000 confirmed cases and a rapidly escalating death toll (de Souza et al., 2020; Hallal et al., 2020).

Regardless of the frequent warnings and inclusion of pandemics in national threat assessments, many governments were unprepared to handle the COVID-19 crisis effectively (Boin et al., 2020). The novel nature of the virus posed significant challenges, as the critical information needed to design effective interventions—such as transmission dynamics, symptomatology, incubation periods, recovery and mortality rates, and vulnerable population segments—became clear only after extensive study and practical experience (Boin et al., 2020; Capano et al., 2020). Thus, the initial global response saw a variety of strategies, ranging from stringent lockdowns and travel bans to massive testing and contact tracing efforts, as well as preparing hospitals’ infrastructure and staff for expected surges in patient numbers. This included mobilizing additional healthcare workers (HCWs), acquiring medical supplies and equipment, postponing elective procedures, and leveraging telemedicine to keep non-COVID-19 cases away from health services (Hollander & Carr, 2020; Ranney et al., 2020; Haldane et al., 2021; Benítez et al., 2020).

Even though some countries, like New Zealand and South Korea, implemented early and aggressive measures that significantly curbed the spread of the virus (Baker et al., 2020; Kim et al., 2020), in Brazil, the response to the pandemic was marked by significant challenges and controversies. Initially, there was a lack of coordination between federal and state governments, leading to the inconsistent implementation of public health measures. The federal government often downplayed the severity of the virus, while state governors imposed varying degrees of social distancing measures (Benítez et al., 2020; Hallal & Victora, 2021; Fonseca et al., 2021; Rocha et al., 2021; Aquino et al., 2020). HCWs and researchers called for more robust actions, including widespread testing, increased healthcare capacity, and clear public communication strategies (Hallal & Victora, 2021).

Despite the implementation of these various strategies to curb the spread of COVID-19 and the preparation of hospitals’ infrastructure and staff for expected surges in patient numbers, the pandemic had profound effects on the mental health of HCWs. HCWs faced significant levels of anxiety, depression, and psychological distress (Lai et al., 2020; Pappa et al., 2020) due to social isolation, prolonged working hours, lack of adequate personal protective equipment (PPE), changes in job functions, and fear of infection and of taking the infection home to their family and loved ones (Menon et al., 2022; Mediavilla et al., 2021; Shanafelt et al., 2020; Portugal et al., 2022). The intense pressure on the healthcare system and the rapid mobilization of additional HCWs also led to a situation where even inadequately trained HCWs had to be deployed in critical roles, further exacerbating these issues (Perraud et al., 2022; Tauber et al., 2021; Koontalay et al., 2021). In Brazil, the impact on HCWs’ mental health was further compounded by the high number of cases and deaths, while the shortage of PPE and the high rates of infection among HCWs (de Lima Osório et al., 2023) may also have led to a heightened sense of vulnerability and fear.

Although the mental health impact of the COVID-19 pandemic on HCWs was severe, they were not the only ones exposed to the mental health risks posed by the interaction between the pandemic and COVID-related work changes. Non-healthcare workers (NHCWs) also faced disrupted working conditions due to the abrupt transition to remote work, which introduced challenges such as a lack of a clear separation between work and personal life, extended working hours, and reduced social interactions, among other difficulties (Xiao et al., 2021). Meanwhile, those unable to work remotely experienced a heightened likelihood of SARS-CoV-2 exposure and infection, along with the associated fear of contagion (Faghri et al., 2021; Acke et al., 2022). Furthermore, all workers faced broader concerns, such as job insecurity and potential income losses, which were associated with adverse mental health outcomes (Ruengorn et al., 2021).

Despite the significant body of research on the mental health of HCWs during the COVID-19 pandemic, studies specifically focused on NHCWs remain comparatively less frequent, as most research has targeted HCWs, children and adolescents, vulnerable groups (the elderly, pregnant women, and patients with chronic diseases), or the general population (which includes but is not limited to NHCWs). This gap in the literature highlights the need for further exploration, particularly given the complexity of the Brazilian context, which presents distinct socio-political and healthcare challenges that could influence mental health outcomes among both HCWs and NHCWs. Additionally, focusing on the early months of the pandemic is essential to provide crucial information on the initial impacts and coping mechanisms of these workers, as this period is critical for implementing potential future timely and optimized interventions to mitigate the burden of pandemic events on mental health. Therefore, the aim of this study was to evaluate the prevalence of psychological distress and to identify its associated factors among Brazilian HCWs and NHCWs during the initial stage of the COVID-19 pandemic. Additionally, the study examined a less explored aspect of mental health during the COVID-19 pandemic—the creation of interaction terms to assess whether the predictor variables interact with the experience of living with an infected family member to influence the occurrence of psychological distress.

## 2. Materials and Methods

This cross-sectional study is part of a broader international initiative coordinated from Spain, involving researchers across 16 countries in Latin America, Europe, Africa, and Asia. Data collection in Brazil took place from 23 April to 30 May 2020. To adhere to the crucial social distancing measures aimed at controlling the COVID-19 pandemic, address mobility restrictions, and protect the research team, participants were recruited digitally. Invitations were distributed via email, press releases, and social media platforms such as WhatsApp, Facebook, Instagram, Twitter, and LinkedIn. Participation was entirely voluntary, with no incentives or compensation offered. Prior to completing the survey, each participant provided virtual informed consent.

### 2.1. Sampling

The study utilized a snowball sampling method with multiple entry points, whereby respondents were encouraged to invite others to participate after completing the survey. Snowball sampling is often used in studies targeting populations that are considered hard-to-reach or hidden, where selecting participants who meet the specific eligibility criteria is challenging. Given the lack of a national registry for Brazilian workers and the high percentage of informal employment (41.6% in 2019), this method was considered appropriate for participant recruitment. Although HCWs are generally not considered a “hard-to-reach” or “hidden” population, the pandemic introduced unique obstacles such as stigmatization, frequent changes in work locations, and sick leave due to suspected COVID-19 infections. These challenges complicated efforts to identify and recruit these workers. Therefore, snowball sampling was selected for its effectiveness in overcoming these barriers and ensuring robust participation, particularly among frontline workers.

### 2.2. Research Instrument

Data collection was conducted through a self-administered online questionnaire, the Emotional Impact Questionnaire COVID-19 Brazil (EIQ-BR), which was open to anyone interested in participating. EIQ-BR was adapted from a validated questionnaire originally developed for the Spanish population. Details regarding the development, validation, and psychometric properties of the original version can be found in earlier articles (Gómez-Salgado et al., 2021). The translation and cultural adaptation of the EIQ-BR followed the guidelines outlined by Beaton et al. for ensuring the accuracy and relevance of cross-cultural questionnaire adaptations (Beaton et al., 2000). This process included five key steps: (1) forward translation, (2) synthesis of the translated versions, (3) backward translation, (4) evaluation by an expert committee and proposal of the prefinal version, and (5) testing of the prefinal version. The further specifics of this process are documented in another publication (Alonso et al., 2024).

EIQ-BR’s final version comprised 147 questions distributed across 11 sections, each addressing distinct aspects relevant to the study. These sections encompassed sociodemographic characteristics, occupational factors, health-related aspects, COVID-19 knowledge, COVID-19 contact history, self-reported COVID-19 symptoms, COVID-19 risk perception, preventive behaviors, sense of coherence, work engagement, and psychological distress.

It is important to note that commonly used and validated instruments—such as the Sense of Coherence Scale, Utrecht Work Engagement Scale, and General Health Questionnaire—were included in the original EIQ to expedite its validation process and prevent delays in data collection, especially given the urgent need to assess the pandemic’s effects. These instruments had already been translated and validated for use in Brazil, ensuring that the EIQ-BR was both valid and contextually appropriate for the research.

### 2.3. Predictor Variables

#### 2.3.1. Sociodemographic

In this study, a variety of sociodemographic factors were investigated. This included sex, categorized as either male or female; age, measured in full years; and marital status, which was classified into single, married or cohabiting, separated/divorced, and widowed. Educational attainment was also assessed, with categories ranging from high school diploma to advanced degrees such as bachelor’s, specialization, master’s, and PhD. Participants were categorized based on their region of residence within Brazil, including the North, Northeast, Midwest, Southeast, and South.

Additionally, the type of residence was analyzed, distinguishing between apartments with or without balconies, houses with or without backyards, and other types of living arrangements. Other sociodemographic factors considered were whether the participants had children, owned pets, or lived with individuals with various types of disabilities. Specifically, this included living with someone with a physical disability, an intellectual disability, disabilities affecting vision or hearing, or multiple disabilities. The last five variables were recorded using “yes or no” responses.

#### 2.3.2. Occupational

The study also evaluated several occupational variables. These included the major occupational group, categorized as white collar, blue collar, pink collar, and others. White-collar occupations encompassed roles such as scientists, artists, executives, administrative staff, managers, and technicians. Blue-collar jobs included positions like farmers, foresters, fishermen, and those involved in industrial production, repair, and maintenance. Pink-collar roles were associated with the service sector, while the “others” category covered military personnel, police officers, firefighters, and other roles not classified by the Brazilian Occupation Classification Index.

Additional occupational factors assessed were whether individuals were healthcare professionals, with responses categorized as yes or no; their type of employment relationship, including self-employed, civil servant, and private sector employee; and their work arrangement, which could be part time at home, part time not at home, full time at home, full time not at home, or mixed.

The study also examined perceptions regarding workplace conditions using five items: whether the employer provided all the necessary materials for efficient and safe work, workplace conflicts, increased workload, work-related stress, and overall work satisfaction. Each of these aspects was measured on a scale from 1 to 10. For material provision, 1 indicated “disagree completely” and 10 indicated “agree completely”. Workplace conflicts, increased workload, and work-related stress were rated from 1 (“definitely not”) to 10 (“definitely yes”). Lastly, overall work satisfaction was evaluated on a scale where 1 represented “completely dissatisfied” and 10 represented “completely satisfied”.

#### 2.3.3. Health-Related

Similarly, the study investigated several health-related variables. Participants were asked to assess their own health status over the 14 days prior, with the following options: very good, good, fair, poor, and very poor. Other variables included whether participants self-identified as having a disability or a chronic disease, and whether they reported using medication. The study also inquired about the self-reported utilization of healthcare services and any hospitalization history within the 14 days prior. For the last five of these variables, responses were categorized as either “yes” or “no”.

#### 2.3.4. COVID-19 Knowledge

The study examined several variables related to participants’ COVID-19 knowledge. Participants’ information sources were categorized into various groups, including social media and friends/family, traditional platforms (such as newspapers, radio, and television), official platforms (websites of official institutions or scientific societies), and others (e.g., search engines like Google and scientific articles). Responses were further grouped based on the number of sources used, ranging from two to all available sources. Additionally, the clarity and accuracy of information provided by employers about COVID-19 were assessed on a scale from 1 (completely dissatisfied) to 10 (completely satisfied). The study also measured participants’ daily exposure to COVID-19 information, categorized by hours per day, ranging from less than 1 h to more than 8 h. Other variables included whether participants engaged in fact-checking (yes or no) and self-assessments of their knowledge about COVID-19 transmission, preventive measures, symptoms, prognosis, and treatment, using a 1-to-10 scale, with 1 indicating insufficient knowledge and 10 indicating sufficient knowledge.

Additionally, the EIQ-BR included five questions addressing basic COVID-19 knowledge, covering topics such as the incubation period, symptoms, isolation requirements following a positive test, transmission methods, and the transmission period. Participants could respond with “yes”, “no”, or “I do not know” for each question. A composite score for basic COVID-19 knowledge was created by assigning one point for each correct answer, while incorrect answers and “I do not know” responses were assigned zero points.

#### 2.3.5. COVID-19 Contact History

This study also investigated variables related to participants’ COVID-19 contact history. These included whether they lived with a family member who had been infected (with possible responses of yes, no, or not having an infected family member). Additionally, participants were asked if any of their co-workers had been infected. The nature of contact with confirmed COVID-19 cases was assessed by distinguishing between close contact (defined as being within 2 meters for more than 15 min) and casual contact. Participants were also questioned about any contact with people or materials suspected of being infected. For the last four questions, the response options were “yes”, “no”, or “do not know”. Finally, participants indicated whether they had been tested for COVID-19, with a simple yes or no response.

#### 2.3.6. COVID-19 Symptoms

The EIQ-BR also gathered information regarding participants’ perceived COVID-19 symptoms within the 14 days prior, focusing on symptoms such as cough, shortness of breath, fever, sore throat, rhinitis, chills, headache, myalgia, dizziness, and diarrhea. Based on these data, two variables were analyzed: whether participants reported experiencing at least one symptom during this period (with yes or no responses) and the number of symptoms they presented, categorized as none, one, between two and four, between five and seven, and between eight and ten symptoms.

#### 2.3.7. COVID-19 Risk Perception

Similarly, the questionnaire included nine questions designed to assess participants’ perceptions of COVID-19 risk. Each question was rated on a scale from 1 to 10, with 1 indicating “not worried at all” and 10 indicating “very worried”. A cumulative risk perception score ranging from 9 to 90 was created by summing the responses to these questions. Additional variables related to risk perception included self-perception of the risk of COVID-19 infection at work and acceptance of COVID-19 infection as an occupational hazard. These three variables were also rated on a scale from 1 to 10, where 1 signified “definitely not” and 10 signified “definitely yes”. Furthermore, participants were asked whether they thought they might have contracted COVID-19, with response options of “yes”, “no”, and “do not know”.

#### 2.3.8. Preventive Behaviors

The EIQ-BR also assessed engagement in preventive measures through a series of questions with five response options, ranging from “never” to “always”. Participants reported the frequency of specific behaviors, including covering their mouth with their elbow when sneezing or coughing, avoiding the sharing of eating utensils, washing hands with soap and water, using a hydroalcoholic solution to wash hands, washing hands immediately after touching their nose, sneezing, or coughing, washing hands after contact with potentially contaminated objects, wearing a face mask regardless of symptoms, and keeping at least 1.5 m apart from others. Responses were converted into a numerical scale from 1 (never) to 5 (always) and a composite preventive behavior score was calculated by averaging the scores across all eight items. Additionally, the perception of preventive measure effectiveness was evaluated on a scale from 1 (not effective at all) to 10 (very effective).

#### 2.3.9. Sense of Coherence

To assess participants’ sense of coherence, the EIQ-BR utilized the Brazilian Portuguese adaptation of the 13-item Sense of Coherence Scale (SOC-13). The internal consistency of the SOC-13 for the Brazilian Portuguese version was confirmed with a Cronbach’s alpha of 0.81 (Freire et al., 2001). This self-administered instrument included thirteen items rated on a seven-point Likert scale and was divided into three components: comprehensibility, manageability, and meaningfulness (Eriksson & Mittelmark, 2016). For data analysis, the total score on the scale was used, which was calculated by summing the scores from all 13 items, with possible scores ranging from 13 to 91 points. Higher scores indicated a stronger sense of coherence.

#### 2.3.10. Work Engagement

To evaluate work engagement, the EIQ-BR employed the Brazilian Portuguese version of the 9-item Utrecht Work Engagement Scale (UWES-9). The UWES-9 demonstrated strong internal consistency in its Brazilian Portuguese version, with a Cronbach’s alpha of 0.94 (Vazquez et al., 2015). This self-administered instrument consisted of nine items measured on a seven-point Likert scale, encompassing three key dimensions: vigor, dedication, and absorption (Vazquez et al., 2015). For the purpose of data analysis, the mean score across all nine items was calculated, yielding a possible range from 0 to 6 points. Higher mean scores indicated greater levels of work engagement.

### 2.4. Outcome Variable

#### Psychological Distress

To assess psychological distress, the EIQ-BR utilized the Brazilian Portuguese adapted version of the 12-item General Health Questionnaire (GHQ-12). The internal consistency of the Brazilian Portuguese version of the GHQ-12 was strong, with a Cronbach’s alpha of 0.88 (Borges & Argolo, 2002). This self-administered instrument was designed to screen for psychological well-being and identify non-psychotic psychiatric disorders (Goldberg et al., 1997). Each item on the GHQ-12 offered four response options, with the first two scored as zero points and the last two scored as one point. In this study, a cut-off point of 3 was used to determine the prevalence of psychological distress, with scores of 3 or higher indicating its presence. For the analysis of associated factors, psychological distress was treated as a continuous variable.

### 2.5. Data Analysis

Prior to conducting statistical analysis, data screening procedures were performed. Outliers were identified and responses deemed invalid or inconsistent were reviewed and excluded when necessary to maintain data integrity. Variables were coded according to the statistical analysis plan. Additionally, key statistical assumptions—such as normality, homogeneity of variances, and multicollinearity—were assessed as appropriate for each analysis. All variables analyzed in this study, along with their detailed descriptions, how they were handled in the analysis, and the corresponding missing data values, are provided in the Supplementary Materials (Tables S1–S9).

#### 2.5.1. Missing Data

To address the missing data, multiple imputation using chained equations was performed with the “mice” function from the “mice” package in R 4.1.1, employing the predictive mean matching method (van Buuren & Groothuis-Oudshoorn, 2011; van Buuren et al., 2021).

#### 2.5.2. Descriptive Statistics and Group Comparisons

Basic descriptive statistics, including percentage distributions for categorical variables and measures of central tendency and dispersion for numerical variables, were used to provide an overview of the sample’s characteristics. To evaluate differences between healthcare professionals and professionals from other fields, the chi-square test was applied using IBM SPSS 21.0 software. Chi-square tests were followed by Cramér’s V effect size measurement, as the standard error tends to approach zero in large samples, making even small differences statistically significant but of limited clinical and epidemiological relevance. Associations were deemed meaningful when Cramér’s V was ≥0.30, 0.21, 0.17, 0.15, and 0.13 for 1, 2, 3, 4, and ≥5 degrees of freedom, respectively. Additionally, for numerical variables, Mann–Whitney tests followed by the “Impact {X1,X2}” effect size measurement (Lötsch & Ultsch, 2020) were conducted using the “Impact” function from the “ImpactEffect-size” package in R 4.1.1. Differences in numerical variables between healthcare and other professionals were considered of clinical–epidemiological significance when the absolute value of “Impact {X1,X2}” exceeded 0.50.

#### 2.5.3. Regression Models for Psychological Distress

Multiple linear regression models, with the response variable following a Poisson distribution, were employed to examine the relationship between the predictor variables and psychological distress. Initially, we performed simple linear regressions with a Poisson-distributed outcome for each predictor variable separately. Variables with a *p*-value < 0.20 in these univariate analyses were selected for inclusion in the multiple regression model. Subsequently, a multiple linear regression model was fitted, using the smallest deviance value as the criterion for final model selection. The final model retained only the variables that were statistically significant (*p* < 0.05).

Then, the interaction terms between living with infected family members and the other variables retained in the final model were created and introduced into the multiple regression model to identify whether effect modification was present; again, the best-fitted model was chosen based on the lowest deviance value.

Deviance is a measure defined as twice the difference between the logarithm of the likelihood function of the saturated model (the model with the maximum number of parameters) and the logarithm of the likelihood function of the model under investigation. Therefore, two models, one denoted as M0 (with q parameters and deviance D0) and the other as M1 (with p parameters and deviance D1), which have the same distribution and the same link function, but with deterministic components that make M0 a particular case of M1, can be compared to determine which one better describes the data. This comparison can be made by calculating the difference between the deviances of models M0 and M1, denoted as ΔD = D0 − D1, and comparing it to the χ^2^ distribution with (p − q) degrees of freedom. If the value of ΔD falls within the critical region of the chi-square distribution with p-q degrees of freedom, established by the defined significance level α, then the conclusion is to reject M0 in favor of M1 (Dobson & Barnett, 2008).

Finally, the performance of the final model was evaluated by estimating its accuracy, sensitivity, and specificity through a comparison of observed and predicted values.

#### 2.5.4. Ethical Considerations

The study was authorized by the Brazilian National Research Ethics Committee (CAAE 30437120.4.0000.5411, 23 April 2020) and informed consent was obtained from all subjects involved in the study.

## 3. Results

Data from 2903 individuals were analyzed, with 1752 classified as NHCWs and 1151 as HCWs. Detailed characteristics of the participants were previously published (Alonso et al., 2024) in a study that explored the psychological distress faced by Brazilian workers (both NHCWs and HCWs) during the initial phase of the COVID-19 pandemic, while also exhaustively describing key factors that may have influenced their mental health during this time.

Of the 52 predictor variables examined, 20 showed no statistically significant differences between NHCWs and HCWs (Table 1). Among the 32 variables with significant differences, only 7 demonstrated clinical–epidemiological relevance, as indicated by their effect size. These seven variables are presented in Table 2.

Regarding the dependent variables, 72.6% (95% CI 70.1–74.2%) of the participants had a GHQ-12 score equal to or greater than three, indicating psychological distress. When stratified by occupational group, 72.8% (1275 individuals) of NHCWs and 72.4% (833 individuals) of HCWs met the criteria for psychological distress. There was no statistically significant difference between the two groups (*p* = 0.812).

In the initial simple linear regressions, 28 variables yielded a *p*-value < 0.20 and were thus included in the multiple regression modeling process. Table 3 presents the results associated with these variables. The findings for variables that did not meet the significance criteria are detailed in the Supplementary Materials (Table S10).

In the multiple linear regression analysis, only experiencing more work-related stress, sense of coherence, and living with an infected family member retained statistical significance, with *p*-values lower than 0.05, and thus remained in the final model. The results for these variables are presented in Table 4.

The analysis of the interaction effects between experiencing more work-related stress and living with infected family members on psychological distress, as well as between a sense of coherence and living with infected family members, revealed two key findings. Firstly, while psychological distress decreased with a higher sense of coherence, this effect became more subtle and less epidemiologically relevant among those who did not live with or have infected family members. Secondly, psychological distress increased together with work-related stress; however, this association ceased to be significant for individuals who had no infected family members (Table 5). This model presented 81.7% accuracy, 88.0% sensitivity, and 65.0% specificity when its predicted values were compared with the actual observations.

## 4. Discussion

Our findings revealed a high prevalence of psychological distress (72.6%), defined as an individual’s response to both external and internal stressors, often manifesting through a combination of psychological symptoms such as stress, depression, and anxiety (Ridner, 2004). Notably, no significant difference in psychological distress was observed between NHCWs and HCWs. In contrast, 32 predictor variables exhibited statistically significant differences between these groups, and of these, only 7 were considered of clinical–epidemiological relevance. This result supports the decision to evaluate effect sizes, as they helped distinguish meaningful differences from those of limited practical importance. Emphasizing the clinically relevant outcomes provided a more comprehensive interpretation, moving beyond statistical significance toward a deeper understanding of the data.

The prevalence of psychological distress observed in this study was notably higher than that reported in numerous other countries. Several factors may have contributed to such a high prevalence of psychological distress, including the greater percentage of female participants, the significant proportion of remote workers, the perception of an increased workload, and the high incidence of chronic diseases among the participants. Additionally, more than half of Brazilians expressed low or no confidence in the government and considered the country’s pandemic response inadequate and inefficient. Since all these factors have been explored in depth in a previous publication (Alonso et al., 2024). We will only focus on the lack of difference in the occurrence of psychological distress between NHCWs and HCWs.

The absence of a significant difference in the prevalence of psychological distress between NHCWs and HCWs found in this study is supported by the findings of three meta-analyses. The first meta-analysis, published in 2020, included 62 studies involving 162,639 participants from 17 countries, and reported that the prevalence of anxiety, depression, and distress was comparable between HCWs and the general population, with overlapping 95% confidence intervals (Luo et al., 2020). The second meta-analysis, published in 2021, included 206 studies with a sample size of 721,244, and found similar prevalences of anxiety, depression, post-traumatic stress, and insomnia among HCWs and the general population (Phiri et al., 2021). The third meta-analysis, published in 2022, incorporated a total of 158 studies involving 880,352 individuals from 41 countries, and similarly indicated that the pooled prevalence of depression, anxiety, and stress was similar among HCWs and the general population, though healthcare workers reported slightly higher rates of anxiety and depression and lower levels of well-being (Blasco-Belled et al., 2024). Notably, subgroup analyses within this study revealed that the impact of COVID-19 on mental health was more pronounced in countries with fewer resources, such as for HCWs (for anxiety), hospital beds (for stress), and health expenses (for anxiety and stress) (Blasco-Belled et al., 2024). Consequently, it is not surprising that psychological distress was higher among the general population compared to healthcare workers in regions of America and Southeast Asia (Blasco-Belled et al., 2024).

Conversely, a fourth meta-analysis, published in 2020, analyzed data from 50 studies with 171,571 participants from 5 countries, and also found similar prevalences of stress, depression, and anxiety across HCWs and the general population. However, it reported a significantly higher prevalence of psychological distress among HCWs (41%) compared to the general population (26%). It is important to note that most of the included studies (46 out of 50) were conducted in China, with one study each in Iran, Italy, Singapore, and Vietnam (Krishnamoorthy et al., 2020).

Despite the higher prevalence of SARS-CoV-2 infection among HCWs compared to the general population (Gómez-Ochoa et al., 2021), along with increased workloads (Doleman et al., 2023), and the unique work-related emotional demands they face (Portoghese et al., 2021; Mosheva et al., 2021)—all of which can negatively impact mental health—previous research has shown that good socioeconomic status and higher levels of education served as protective factors for mental health during the COVID-19 pandemic (Kunzler et al., 2021), as did health literacy (Hermans et al., 2021). In general, and as seen in our study, HCWs tend to have better socioeconomic conditions and higher educational attainment compared to other occupational groups.

These factors could act as protective elements by providing greater access to health resources (such as health insurance and healthcare services), financial resources (such as savings and investment accounts, as well as access to personal loans or lines of credit), and decent, stable work, which ensures a predictable income, essential benefits, and better working conditions, ultimately mitigating the adverse effects of some pandemic-related stressors. Higher education levels may also contribute to enhanced coping mechanisms, problem-solving skills, and greater awareness of mental health, all of which help to reduce the impact of distressing experiences. Consequently, these factors may have helped HCWs to mitigate psychological distress and manage stressors more effectively, contributing to their mental well-being during the period covered by the study.

Although NHCWs did not face the specific mental health risks of HCWs, some of them were not working from home, increasing their risk of SARS-CoV-2 infection. In contrast, NHCWs working remotely encountered a unique set of stressors—poor working conditions, perceptions of increased workloads, unclear job expectations, and work–life conflict—that were relatively unfamiliar before the pandemic. Additionally, remote workers often experienced feelings of diminished value within their organizations, as well as a lack of efficient social support systems (Costin et al., 2023). Therefore, remote work also presented significant and unique challenges that affected mental well-being, including heightened anxiety, depression, and emotional exhaustion (Costin et al., 2023). These factors may explain the lack of significant difference in psychological distress between NHCWs and HCWs found in our study, as well as in the meta-analyses cited, where both groups presented similar prevalences of psychological distress during the pandemic.

Regarding the predictor variables that showed statistically significant differences between NHCWs and HCWs and were also deemed of clinical–epidemiological relevance, six of the seven were directly related to occupational factors—educational attainment, major occupational group, work arrangement, close contact with confirmed infected individuals, contact with suspected infected materials or people, and acceptance of COVID-19 as an occupational hazard. These differences were expected, given the professional distinctions between NHCWs and HCWs. However, the disparity in educational attainment between the two groups may reflect broader Brazilian socioeconomic inequalities, where disparities in education and occupational status remain significant (Harris, 2022). Finally, the variation based on the regional distribution of participants likely resulted from the snowball sampling method used. As the research team was largely composed of healthcare professionals, their networks were likely more regionally diverse, particularly in healthcare-related fields, compared to other occupational sectors.

Moving to the relationship between sense of coherence and psychological distress, as originally conceptualized by Antonovsky (1996), sense of coherence is consistently shown as a protective factor against stress, as being associated with less psychological distress, and as and mediating the relationship between anxiety and depression (Danioni et al., 2023; Barni et al., 2020; Padmanabhanunni, 2022). Sense of coherence is composed of the following interrelated components: comprehensibility, manageability, and meaningfulness (Antonovsky, 1996). Thus, a strong sense of coherence enables individuals to recognize a stressful situation as structured, consistent, and clear (comprehensibility), perceive the resources that are available to cope with the demands posed by the situation (manageability), and apply the appropriate resources to develop an effective response to it (meaningfulness) (Antonovsky, 1996).

Our study’s finding that during the initial stages of the COVID-19 pandemic psychological distress decreased with a higher sense of coherence aligns with previous research (Berger-Estilita et al., 2022; Gómez-Salgado et al., 2022; Schäfer et al., 2022). However, this effect was less pronounced when the surrounding stressors, such as family members infected with COVID-19, were absent, making the association weaker in those cases. It has already been shown that sense of coherence’s ability to protect against the negative effects of stressful experiences might depend on the severity of the events, being protective only in situations with high stress (Lutgendorf et al., 1999), such as living with an infected family member during a pandemic event. This suggests that interventions aimed at increasing sense of coherence might be useful for preventing psychological distress, especially in high-stress occupations and/or events.

Our findings also revealed a positive association between work-related stress—defined as the harmful physical and emotional reactions that arise when the demands of the job exceed the worker’s capabilities, resources, or needs (National Institute for Occupational Safety and Health, 2008)—and psychological distress. This relationship between occupational stress and mental health challenges has been documented in previous research (Foti et al., 2023; Ravikumar, 2023; Nishihara et al., 2022). However, in our study, this association lost significance in the absence of family members infected with COVID-19, suggesting that the effect of family-related stressors, such as caring for an infected family member, may exacerbate the impact of work-related stress on mental health.

Workers experiencing high levels of stress from job demands may already face diminished emotional and cognitive resources to manage work demands (Foti et al., 2023). When this stress is intensified by a significant life event, like living with a family member infected with COVID-19, workers’ ability to cope further diminishes, leading to worsened mental health outcomes. This highlights the interplay between work-related stress and family obligations, a conflict commonly known to result in psychological distress (Mopkins, 2022). In fact, role conflicts—especially between work and heavy family caregiving responsibilities (Yucel & Fan, 2023)—are well-documented predictors of poor mental health outcomes, often seen in individuals balancing competing demands from their professional and family lives (Mopkins, 2022).

Research consistently shows that caregiving has a negative impact on emotional well-being. Caregivers are more likely to experience emotional exhaustion, anxiety, depression, and physical health issues due to the added burden of caring for a loved one (Honda et al., 2014).

### 4.1. Limitations and Strengths

While our findings add to the understanding of psychological distress among workers during the initial stages of a pandemic event, some limitations should be considered when interpreting the results. The use of snowball sampling may have affected the representativeness of the sample. This technique often leads to the recruitment of participants with similar social networks, potentially introducing selection bias and limiting diversity in the sample. As a result, certain subgroups, particularly those with less access to social connections or differing characteristics, might have been underrepresented. While efforts were made to mitigate this by recruiting individuals with diverse educational, socioeconomic, and working backgrounds in the first wave, and asking them to contact only three to five new recruits in the subsequent wave, and so forth, it remains difficult to ensure a fully representative sample.

The use of both snowball sampling and online data collection methods was necessary due to the legal and ethical constraints aimed at reducing COVID-19 transmission and safeguarding the research team. It is worth noting that many studies examining psychological distress during the COVID-19 pandemic also employed snowball sampling and online surveys, as in-person recruitment was not feasible under the public health guidelines. While this approach was practical under the circumstances, it remains a limitation that could have introduced bias and affected the generalizability of the findings.

Moreover, it is recognized that individuals with severe or pre-existing mental health conditions are less likely to participate in online surveys (Pierce et al., 2020). As a result, although the prevalence reported in this study is high, it may still underestimate the true extent of psychological distress among Brazilian workers during the studied period. This limitation should be considered when interpreting the broader impact of the pandemic on workers’ mental health.

Another limitation of this study lies in the cross-sectional design of the study, since it only captures data at a single point in time. This makes it difficult to establish causal relationships between the variables analyzed. In particular, reverse causality may be an issue, where the direction of the relationship between psychological distress and other variables, such as work-related stress, cannot be definitively determined. Therefore, caution is required when interpreting these associations, as the study design limits the ability to make causal inferences.

Although the study faced certain limitations, it presented several notable strengths, including a large, geographically diverse sample and the use of internationally validated instruments, which enhanced data reliability and comparability. Additionally, the robust variable selection approach, using deviance to evaluate model fit, minimized overfitting, enhanced interpretability, and ensured a balance between model fit and parsimony.

### 4.2. Implications

The high prevalence of psychological distress among Brazilian workers, observed in both HCWs and NHCWs, highlights the need for targeted interventions to support mental health in occupational settings during pandemic events. Nearly three-quarters of participants reported psychological distress, with higher levels of work-related stress associated with increased psychological distress. Therefore, organizations should implement policies that promote well-being, including mental health support programs, workload management strategies, and adequate work environment and safety equipment. Specific measures should address the challenges of remote work, such as providing all necessary equipment, offering stipends or reimbursements for work-related expenses, and setting clear, measurable goals with realistic deadlines. It is also essential to establish boundaries between work and personal time by setting fixed hours and respecting workers’ time off.

In addition to workplace interventions, this study highlights the protective role of a higher sense of coherence in reducing psychological distress. Occupational interventions that strengthen its components—such as the clear communication of policies, goals, and expectations (enhancing comprehensibility), providing adequate equipment (reinforcing manageability), and cultivating a sense of purpose and meaning at work (promoting meaningfulness)—could mitigate distress. However, our findings indicate that both the protective effect of a strong sense of coherence and the increase in psychological distress due to work-related stress were influenced by family members being infected with COVID-19. This suggests that personal stressors can weaken sense of coherence’s protective role while amplifying the detrimental effects of work-related stress on mental health.

These findings point to broader implications for pandemic preparedness, both in Brazil and globally. Future public health policies should prioritize measures that promote an adequate work environment, ensure access to mental health services, and support both workers and their families to address the combined burden of occupational and family-related stressors. This approach will ensure that workers’ mental health is integrated into emergency response frameworks.

## 5. Conclusions

In conclusion, a total of 2903 Brazilian workers, 1752 NHCWs and 1151 HCW, participated in the study. The prevalence of psychological distress, defined as a GHQ-12 score equal to or greater than three, was 72.6% (95% CI 70.1–74.2%), without any statistically significant difference between NHCWs and HCWs. Regarding psychological distress’ associated factors, our results show that psychological distress decreased with a higher sense of coherence. However, this effect was less pronounced when the participants did not have family members infected with COVID-19. While higher levels of work-related stress increased psychological distress, this association also lost significance in the absence of family members infected with COVID-19. These findings highlight the complex interplay of stressors and social circumstances in predicting psychological outcomes, as well as the importance of addressing both work- and family-related stressors when considering interventions to support workers’ mental health, particularly during crises like the COVID-19 pandemic.

## Figures and Tables

**Table 1:** Variables that showed no statistically significant differences between non-healthcare workers and healthcare workers.

Variable	*p*-Value
Living with someone with a physical disability	0.676
Living with someone with an intellectual disability	0.478
Living with someone with a visual, auditive, or multiple disabilities	0.801
Employer provided all materials and means necessary to work efficiently	0.307
Experienced an increase in workload	0.172
Current work satisfaction	0.111
Self-perceived health status	0.204
Self-identifying as having a disability	0.348
Self-identifying as having a chronic disease	0.402
Self-reported healthcare utilization	0.423
Self-reported hospitalization history	0.471
Clarity and accuracy of employer information	0.747
Fact-checking	0.564
Self-perceived COVID-19 transmission knowledge	0.126
Self-perceived COVID-19 prognosis knowledge	0.416
Self-perceived COVID-19 treatment knowledge	0.536
COVID-19 basic knowledge score	0.417
Number of COVID-19 symptoms presented	0.596
COVID-19 risk perception score	0.060
Perceived effectiveness of preventive measures	0.180

**Table 2:** Variables that presented clinically and epidemiologically significant differences between non-healthcare workers (NHCWs) and healthcare workers (HCWs).

Variable	NHCWs		HCWs		*p*-Value	ES
	n or Median	% or IQR	n or Median	% or IQR		
Educational attainment						
High school	255	14.6	37	3.2	<0.001	0.27
Bachelor	561	32.0	208	18.2		
Specialization	420	24.0	393	34.1		
Master	292	16.6	265	23.0		
PhD	224	12.8	248	21.5		
Brazilian region of residence						
North	11	0.6	18	1.6	<0.001	0.13
Northeast	62	3.5	84	7.3		
Midwest	113	6.4	120	10.4		
Southeast	1379	78.8	777	67.5		
South	187	10.7	152	13.2		
Major occupational group						
White collar	1128	64.4	940	81.7	<0.001	0.19
Blue collar	109	6.2	14	1.2		
Pink collar	5	0.3	0	0.0		
Others	510	29.1	197	17.1		
Work arrangement						
Part time at home	341	19.5	134	11.6	<0.001	0.40
Part time not at home	165	9.4	219	19.0		
Full time at home	941	53.7	259	22.5		
Full time not at home	200	11.4	454	39.4		
Mixed	105	6.0	85	7.4		
Close contact with confirmed infected person						
Yes	26	1.4	138	12.0	<0.001	0.22
No	870	49.7	483	42.0		
Do not know	856	48.9	530	46.0		
Contact with suspected infected materials or people						
Yes	65	3.7	190	16.5	<0.001	0.22
No	331	18.9	140	12.2		
Do not know	1356	77.4	821	71.3		
Acceptance of COVID-19 infection as an occupational hazard	1	4	6	8	<0.001	1.29

Key terms: n indicates the number of participants, IQR refers to the interquartile range, and ES represents the effect size.

**Table 3:** Predictor variables associated with psychological distress in the initial simple linear regressions.

Variable	PR	95% CI	*p*-Value
Sex (ref. female)			
Male	0.84	0.76–0.93	0.001
Age	0.99	0.99–0.99	<0.001
Marital status (ref. single)			
Married/cohabiting	0.90	0.82–0.98	0.018
Separated/divorced	0.90	0.77–1.06	0.203
Widowed	0.90	0.57–1.43	0.665
Children (ref. no)			
Yes	0.90	0.82–0.98	0.014
Work arrangement (ref. part time at home)			
Part time not at home	0.94	0.80–1.10	0.409
Full time at home	0.98	0.87–1.11	0.790
Full time not at home	0.96	0.84–1.10	0.563
Mixed	0.85	0.70–1.05	0.133
Employer provided all materials and means to work efficiently	0.97	0.96–0.99	<0.001
Employer provided all materials and means to work safely	0.98	0.97–1.00	0.017
Experienced more conflicts at work	1.03	1.01–1.04	<0.001
Workload increase	1.02	1.01–1.03	0.003
Work-related stress increase	1.08	1.06–1.09	<0.001
Current work satisfaction	0.94	0.93–0.96	<0.001
Self-perceived health status (ref. very good)			
Good	0.94	0.48–1.83	0.862
Fair	0.88	0.47–1.65	0.694
Poor	0.75	0.40–1.40	0.372
Very poor	0.59	0.32–1.11	0.100
Self-reported healthcare utilization (ref. no)			
Yes	1.11	0.95–1.29	0.195
Clarity and accuracy of employer information	0.98	0.97–0.99	0.021
Hours per day exposed to COVID-19 information (ref. up to 1 h)			
>1 up to 4 h	1.07	0.96–1.20	0.238
>4 up to 8 h	1.15	1.01–1.32	0.042
>8 h	1.18	1.02–1.37	0.029
Self-perceived COVID-19 prognosis knowledge	0.99	0.97–1.01	0.194
Self-perceived COVID-19 treatment knowledge	0.98	0.96–0.99	0.008
COVID-19 basic knowledge score	0.95	0.90–1.00	0.050
Living with an infected family member (ref. yes)			
No	1.69	1.46–1.96	<0.001
Have not had an infected family member	2.30	2.05–2.57	<0.001
Any co-worker infected (ref. yes)			
No	0.92	0.81–1.04	0.168
Do not know	0.98	0.86–1.11	0.757
Contact with suspected infected materials or people (ref. yes)			
No	0.99	0.84–1.18	0.932
Do not know	0.88	0.76–1.02	0.082
Tested for COVID-19 (ref. yes)			
No	1.14	0.94–1.38	0.188
Number of COVID-19 symptoms presented (ref. none)			
One	1.22	1.06–1.41	0.007
Between two and four	1.35	1.19–1.53	<0.001
Between five and seven	1.55	1.32–1.82	<0.001
Between eight and ten	1.76	1.15–2.69	0.009
COVID-19 risk perception score	1.02	1.01–1.02	<0.001
Self-perception of work as a risk for COVID-19	1.01	1.00–1.02	0.134
Perceived effectiveness of preventive measures	0.97	0.95–0.99	0.032
Sense of coherence	0.99	0.98–0.99	<0.001
Work engagement	0.87	0.84–0.90	<0.001

Key terms: PR represents the prevalence ratio and CI refers to the confidence interval.

**Table 4:** Predictor variables associated with psychological distress in the final multiple regression model.

Variable	PR	95% CI	*p*-Value
Work-related stress increase	1.04	1.02–1.05	<0.001
Living with an infected family member (ref. yes)			
No	1.49	1.28–1.73	<0.001
Have not had an infected family member	1.82	1.60–2.06	<0.001
Sense of coherence	0.99	0.96–0.99	0.024

Key terms: PR represents the prevalence ratio and CI refers to the confidence interval.

**Table 5:** The interaction effects of living with an infected family member, work-related stress, and sense of coherence on psychological distress.

Variable	PR	95% CI	*p*-Value
Sense of coherence X Living with an infected family member			
Sense of coherence X Living with an infected family member	0.989	0.987–0.991	<0.001
Sense of coherence X Not living with an infected family member	0.994	0.991–0.995	<0.001
Sense of coherence X Not having an infected family member	0.998	0.996–0.999	0.044
Work stress increase X Living with an infected family member			
Work stress increase X Living with an infected family member	1.086	1.057–1.115	<0.001
Work stress increase X Not living with an infected family member	1.056	1.025–1.087	<0.001
Work stress increase X Not having an infected family member	1.014	0.997–1.031	0.116

Key terms: PR represents the prevalence ratio and CI refers to the confidence interval.

## Data Availability

The original contributions presented in this study are included in the article/Supplementary Materials. Further inquiries can be directed to the corresponding author.

## Supplementary Materials

The following supporting information can be downloaded at: https://www.mdpi.com/article/10.3390/bs15030358/s1. Tables S1–S10 (Table S1: Sociodemographic variables description, type, categories/scale, and number of missing data; Table S2: Occupational variables description, type, categories/scale, and number of missing data; Table S3: Health-related variables description, type, categories/scale, and number of missing data; Table S4: COVID-19 knowledge variables description, type, categories/scale, and number of missing data; Table S5: COVID-19 contact history variables description, type, categories/scale, and number of missing data; Table S6: COVID-19 risk perception variables description, type, categories/scale, and number of missing data; Table S7: Sense of coherence variables description, type, categories/scale, and number of missing data; Table S8: Work engagement variables description, type, categories/scale, and number of missing data; Table S9: Psychological distress variables description, type, categories/scale, and number of missing data; and Table S10: Prevalence ratio, confidence intervals, and *p*-values estimated in the simple linear regression models for psychological distress among Brazilian workers).

## Abbreviations

The following abbreviations are used in this manuscript:

HCW	Healthcare workers
PPE	Personal protective equipment
NHCW	Non-healthcare workers
EIQ-BR	Emotional Impact Questionnaire COVID-19 Brazil
SOC-13	Sense of Coherence Scale
UWES-9	Utrecht Work Engagement Scale
GHQ-12	General Health Questionnaire
