# Supplementary material for: Workers’ Psychological Distress During the Early Months of the COVID-19 Pandemic in Brazil: A Cross-Sectional Study
Source: Behav Sci (Basel). 2025 Mar 13;15(3):358. doi: 10.3390/bs15030358 (PMC11939451; doi:10.3390/bs15030358)
Supplement: Supplementary file 1 [file behavsci-15-00358-s001.zip › behavsci-3475371-supplementary.pdf]

Table S1 – Sociodemographic variables description, type, categories/scale and number of missing data

Variable	Description	Type	Categories/Scale	Missing
Sex	Sex of the participant	Nominal	Male, Female	0
Age	Participant's age in complete years	Numeric	Age in complete years	1
Marital status	Marital status of the participant	Nominal	Single, Married/cohabiting, Separated/divorced, Widowed	0
Educational attainment	Highest educational qualification attained	Ordinal	High school, Bachelor's degree, Specialization, Master's degree, PhD	0
Brazilian region of residence	Geographic region of residence	Nominal	North, Northeast, Midwest, Southeast, South	0
Residence type	Type of residence	Nominal	Apartment with balcony, Apartment without balcony, House with backyard, House without backyard, Other	0
Children	Whether the participant has children	Nominal	Yes, No	0
Pet ownership	Whether the participant owns pets	Nominal	Yes, No	0
Living with someone with physical disability	Whether the participant lives with someone with a physical disability	Nominal	Yes, No	311
Living with someone with intellectual disability	Whether the participant lives with someone with an intellectual disability	Nominal	Yes, No	327
Living with someone with visual, auditive, or multiple disabilities	Whether the participant lives with someone with visual, auditive, or multiple disabilities	Nominal	Yes, No	260

Table S2 – Occupational variables description, type, categories/scale and number of missing data

Variable	Description	Type	Categories/Scale	Missing
Major occupational group	Classification of the participant's job	Nominal	White-collar, Blue-collar, Pink-collar, Others	0
Healthcare professional	Whether the participant is a healthcare professional	Nominal	Yes, No	0
Employment relationship	Type of employment relationship	Nominal	Self-employed, Civil servant, Private sector employee	0
Work arrangement	Type of work arrangement	Nominal	Part-time at home, Part-time not at home, Full-time at home, Full-time not at home, Mixed	0
Employer provided all materials and means to work efficiently	Whether the employer provided all materials for efficient work	Numeric	1 (disagree completely) to 10 (agree completely)	0
Employer provided all materials and means to work safely	Whether the employer provided all materials for safe work	Numeric	1 (disagree completely) to 10 (agree completely)	0
Experienced more conflicts at work	Whether the participant experienced more work conflicts	Numeric	1 (definitely not) to 10 (definitely yes)	0
Workload increase	Whether the participant experienced an increase in workload	Numeric	1 (definitely not) to 10 (definitely yes)	0
Work stress increase	Whether the participant experienced more work-related stress	Numeric	1 (definitely not) to 10 (definitely yes)	0
Current work satisfaction	Participant's satisfaction with current work	Numeric	1 (completely dissatisfied) to 10 (completely satisfied)	0

Table S3 – Health-related variables description, type, categories/scale and number of missing data

Variable	Description	Type	Categories/Scale	Missing
Self-perceived health status	Participant's self-assessment of health over the last 14 days	Ordinal	Very good, Good, Fair, Poor, Very poor	0
Self-identifying as having a disability	Whether the participant identifies as having a disability	Nominal	Yes, No	0
Self-identifying as having a chronic disease	Whether the participant identifies as having a chronic disease	Nominal	Yes, No	0
Self-reporting medication use	Whether the participant uses medication	Nominal	Yes, No	0
Self-reported health care utilization	Whether the participant utilized health care services in the last 14 days	Nominal	Yes, No	0
Self-reported hospitalization history	Whether the participant was hospitalized in the last 14 days	Nominal	Yes, No	0

Table S4 – Covid-19 knowledge variables description, type, categories/scale and number of missing data

Variable	Description	Type	Categories/Scale	Missing
Information sources	Different sources of COVID-19 information used	Nominal	Social media and friends/family, Traditional platforms, Official platforms, Others, Two sources, Three sources, Four sources, All sources	0
Clarity and accuracy of employer information	Participant's assessment of employer's COVID-19 information	Numeric	1 (completely dissatisfied) to 10 (completely satisfied)	0
Hours per day exposed to COVID-19 information	Daily hours exposed to COVID-19 information	Ordinal	Up to 1 hour, >1 up to 4 hours, >4 up to 8 hours, >8 hours	0
Fact-checking	Whether the participant engages in fact-checking COVID-19 information	Nominal	Yes, No	0
Self-perceived COVID-19 transmission knowledge	Self-assessed knowledge of COVID-19 transmission	Numeric	1 (insufficient) to 10 (sufficient)	0
Self-perceived COVID-19 prevention knowledge	Self-assessed knowledge of COVID-19 prevention	Numeric	1 (insufficient) to 10 (sufficient)	0
Self-perceived COVID-19 symptoms knowledge	Self-assessed knowledge of COVID-19 symptoms	Numeric	1 (insufficient) to 10 (sufficient)	0
Self-perceived COVID-19 prognosis knowledge	Self-assessed knowledge of COVID-19 prognosis	Numeric	1 (insufficient) to 10 (sufficient)	0
Self-perceived COVID-19 treatment knowledge	Self-assessed knowledge of COVID-19 treatment	Numeric	1 (insufficient) to 10 (sufficient)	0
COVID-19 basic knowledge score	Summed score of COVID-19 basic knowledge questions	Numeric	Minimum of 0 to maximum of 5	166

Table S5 – COVID-19 contact history variables description, type, categories/scale and number of missing data

Variable	Description	Type	Categories/Scale	Missing
Living with infected family member	Whether the participant lives with a family member that was infected with COVID-19	Nominal	Yes, No, Have not had an infected family member	0
Any co-worker infected	Whether any co-worker was infected with COVID-19	Nominal	Yes, No, Do not know	0
Close contact with confirmed infected person	Being within two meters for more than 15 minutes with someone confirmed to be infected	Nominal	Yes, No, Do not know	0
Casual contact with confirmed infected person	Casual contact with someone confirmed to be infected	Nominal	Yes, No, Do not know	0
Contact with suspected infected materials or people	Contact with people or materials suspected of being infected	Nominal	Yes, No, Do not know	0
Tested for COVID-19	Whether the participant was tested for COVID-19	Nominal	Yes, No	0
Number of COVID-19 symptoms presented	Number of different COVID-19 symptoms presented in the last 14 days	Ordinal	None, One, Between two and four, Between five and seven, Between eight and ten	0

Table S6 – COVID-19 risk perception variables description, type, categories/scale and number of missing data

Variable	Description	Type	Categories/Scale	Missing
COVID-19 risk perception score	Summed score of risk perception questions	Numeric	Minimum of 9 to maximum of 90	0
Self-perception of work as a risk for COVID-19	Participant's assessment of work-related COVID-19 infection risk	Numeric	1 (not worried at all) to 10 (very worried)	0
Acceptance of COVID-19 infection as an occupational hazard	Participant's acceptance of COVID-19 as an inherent occupational hazard	Numeric	1 (definitely not) to 10 (definitely yes)	0
Belief that may have contracted COVID-19	Participants' belief that may have contracted COVID-19	Nominal	Yes, No, Do not know	0
COVID-19 preventive behaviors scale	Frequency of engagement in various preventive behaviors	Numeric	Minimum of 1 to maximum of 5	0
Perceived effectiveness of preventive measures	Participant's perception of COVID-19 preventive measures effectiveness	Numeric	1 (not effective at all) to 10 (very effective)	0

Table S7 – Sense of coherence variable description, type, categories/scale and number of missing data

Variable	Description	Type	Categories/Scale	Missing
Sense of coherence	Sense of Coherence Scale (SOC-13) score	Numeric	Minimum of 13 to maximum of 91	0

Table S8 – Work engagement variable description, type, categories/scale and number of missing data

Variable	Description	Type	Categories/Scale	Missing
Work engagement	Utrecht Work Engagement Scale (UWES-9) score	Numeric	Minimum of 0 to maximum of 6	0

Table S9 – Psychological distress variable description, type, categories/scale and number of missing data

Variable	Description	Type	Categories/Scale	Missing
Psychological distress	General Health Questionnaire (GHQ-12) score	Numeric	Minimum of 0 to maximum of 12	0

Table S10. Prevalence ratio, confidence intervals, and p-values estimated in the simple linear regression models for psychological distress among Brazilian workers

Variable	PR	95% CI	p-value
**Sex (ref. female)**			
Male	0.84	0.76-0.93	0.001
**Age**	0.99	0.99-0.99	<0.001
**Marital status (ref. single)**			
Married/cohabiting	0.90	0.82-0.98	0.018
Separated/divorced	0.90	0.77-1.06	0.203
Widowed	0.90	0.57-1.43	0.665
**Educational attainment (ref. high school)**			
Bachelor's degree	1.00	0.85-1.17	0.983
Specialization	0.97	0.83-1.13	0.700
Master's degree	1.01	0.86-1.20	0.869
PhD	1.01	0.85-1.20	0.902
**Brazilian region of residence (ref. North)**			
Northeast	0.92	0.59-1.45	0.731
Midwest	0.94	0.61-1.45	0.766
Southeast	0.91	0.60-1.38	0.660
South	0.91	0.60-1.40	0.684
**Residence type (ref. apartment with balcony)**			
Apartment without balcony	0.97	0.84-1.11	0.652
House with backyard	0.95	0.85-1.06	0.342
House without backyard	0.96	0.80-1.15	0.635
Other	0.94	0.74-1.19	0.583
**Children (ref. no)**			
Yes	0.90	0.82-0.98	0.014
**Pet ownership (ref. no)**			
Yes	1.00	0.92-1.09	0.996
**Living with someone with physical disability (ref. no)**			
Yes	1.07	0.84-1.36	0.603
**Living with someone with intellectual disability (ref. no)**			
Yes	1.00	0.79-1.26	0.994
**Living with someone with visual, auditive, or multiple disabilities (ref. no)**			
Yes	1.02	0.89-1.16	0.783
**Major occupational group (ref. white-collar)**			
Blue-collar	0.984	0.75-1.27	0.905
Pink-collar	1.052	0.84-1.30	0.646
Others	0.984	0.87-1.09	0.725
**Healthcare professional (ref. no)**			
Yes	0.99	0.91-1.09	0.901
**Employment relationship (ref. self-employed)**			
Civil servant	1.03	0.92-1.15	0.624
Private sector employee	1.01	0.90-1.14	0.815
**Work arrangement (ref. part-time at home)**			
Part-time not at home	0.94	0.80-1.10	0.409
Full-time at home	0.98	0.87-1.11	0.790
Full-time not at home	0.96	0.84-1.10	0.563
Mixed	0.85	0.70-1.05	0.133
**Employer provided all materials and means to work efficiently**	0.97	0.96-0.99	<0.001
**Employer provided all materials and means to work safely**	0.98	0.97-1.00	0.017
**Experienced more conflicts at work**	1.03	1.01-1.04	<0.001
**Workload increase**	1.02	1.01-1.03	0.003
**Work stress increase**	1.08	1.06-1.09	<0.001
**Current work satisfaction**	0.94	0.93-0.96	<0.001
**Self-perceived health status (ref. very good)**			
Good	0.94	0.48-1.83	0.862
Fair	0.88	0.47-1.65	0.694
Poor	0.75	0.40-1.40	0.372
Very poor	0.59	0.32-1.11	0.100

Key terms: PR represents the prevalence ratio and CI refers to the confidence interval.

Table S10. Prevalence ratio, confidence intervals, and p-values estimated in the simple linear regression models for psychological distress among Brazilian workers (continued)

Variable	PR	95% CI	p-value
**Self-perceived health status (ref. very good)**			
Good	0.94	0.48-1.83	0.862
Fair	0.88	0.47-1.65	0.694
Poor	0.75	0.40-1.40	0.372
Very poor	0.59	0.32-1.11	0.100
**Self-identifying as having a disability (ref. no)**			
Yes	1.03	0.84-1.27	0.789
**Self-identifying as having a chronic disease (ref. no)**			
Yes	1.04	0.95-1.14	0.405
**Self-reporting medication use (ref. no)**			
Yes	1.02	0.93-1.11	0.699
**Self-reported health care utilization (ref. no)**			
Yes	1.11	0.95-1.29	0.195
**Self-reported hospitalization history (ref. no)**			
Yes	1.16	0.71-1.90	0.552
**Information sources (ref. social media and friends/family)**			
Traditional platforms	0.91	0.64-1.31	0.610
Official platforms	0.85	0.55-1.31	0.467
Others	0.99	0.63-1.55	0.958
Two sources	0.98	0.75-1.28	0.881
Three sources	0.99	0.76-1.28	0.930
Four sources	1.03	0.80-1.33	0.825
All sources	1.06	0.82-1.37	0.670
**Clarity and accuracy of employer information**	0.98	0.97-0.99	0.021
**Hours per day exposed to COVID-19 information (ref. up to 1 hour)**			
>1 up to 4 hours	1.07	0.96-1.20	0.238
>4 up to 8 hours	1.15	1.01-1.32	0.042
>8 hours	1.18	1.02-1.37	0.029
**Fact-checking (ref. no)**			
Yes	1.00	0.84-1.20	0.994
**Self-perceived COVID-19 transmission knowledge**	0.99	0.97-1.01	0.358
**Self-perceived COVID-19 prevention knowledge**	1.00	0.98-1.02	0.803
**Self-perceived COVID-19 symptoms knowledge**	0.99	0.97-1.01	0.300
**Self-perceived COVID-19 prognosis knowledge**	0.99	0.97-1.01	0.194
**Self-perceived COVID-19 treatment knowledge**	0.98	0.96-0.99	0.008
**COVID-19 basic knowledge score**			
**COVID-19 basic knowledge score**	0.95	0.90-1.00	0.050
**Living with infected family member (ref. yes)**			
No	1.69	1.46-1.96	<0.001
Have not had an infected family member	2.30	2.05-2.57	<0.001
**Any co-worker infected (ref. yes)**			
No	0.92	0.81-1.04	0.168
Do not know	0.98	0.86-1.11	0.757
**Close contact with confirmed infected person (ref. yes)**			
No	0.94	0.78-1.14	0.541
Do not know	1.04	0.86-1.26	0.682
**Casual contact with confirmed infected person (ref. yes)**			
No	0.996	0.82-1.20	0.969
Do not know	0.911	0.75-1.09	0.325
**Contact with suspected infected materials or people (ref. yes)**			
No	0.99	0.84-1.18	0.932
Do not know	0.88	0.76-1.02	0.082
**Tested for COVID-19 (ref. yes)**			
No	1.14	0.94-1.38	0.188

Key terms: PR represents the prevalence ratio and CI refers to the confidence interval.

Table S10. Prevalence ratio, confidence intervals, and p-values estimated in the simple linear regression models for psychological distress among Brazilian workers (continued)

Variable	PR	95% CI	p-value
**Number of COVID-19 symptoms presented (ref. none)**			
One	1.22	1.06-1.41	0.007
Between two and four	1.35	1.19-1.53	<0.001
Between five and seven	1.55	1.32-1.82	<0.001
Between eight and ten	1.76	1.15-2.69	0.009
**COVID-19 risk perception score**	1.02	1.01-1.02	<0.001
**Self-perception of work as a risk for COVID-19**	1.01	1.00-1.02	0.134
**Acceptance of COVID-19 infection as an occupational hazard**	0.99	0.98-1.00	0.219
**Belief that may have contracted COVID-19 (ref. yes)**			
No	0.94	0.74-1.20	0.673
Do not know	1.04	0.82-1.33	0.706
**COVID-19 preventive behaviors scale**	1.00	0.91-1.09	0.942
**Perceived effectiveness of preventive measures**	0.97	0.95-0.99	0.032
**Sense of coherence**	0.99	0.98-0.99	<0.001
**Work engagement**	0.87	0.84-0.90	<0.001

Key terms: PR represents the prevalence ratio and CI refers to the confidence interval.
